# Spontaneous Formation of π‐Conjugated Polymeric Colloidal Molecules Through Stepwise Coacervation and Symmetric Compartmentalization

**DOI:** 10.1002/smll.202404934

**Published:** 2024-10-10

**Authors:** Osamu Oki, Shun‐ichiro Noguchi, Sota Nakayama, Hiroshi Yamagishi, Junpei Kuwabara, Takaki Kanbara, Yohei Yamamoto

**Affiliations:** ^1^ Department of Materials Science Institute of Pure and Applied Sciences University of Tsukuba 1‐1‐1 Tennodai, Ibaraki Tsukuba 305‐8573 Japan; ^2^ Institute for Complex Molecular Systems and Laboratory of Macro‐molecular and Organic Chemistry Eindhoven University of Technology Eindhoven 5600 MB The Netherlands

**Keywords:** coacervation, colloidal molecules, liquid‐liquid phase separation, mesophase, self‐assembly

## Abstract

Coacervation, the phase separation of liquid induced by polymeric solutes, sometimes results in the formation of oligomeric clusters of droplets. The morphology of the clusters is non‐uniform because the clustering is a consequence of the random collisions of the drifting droplets. Here we report distinctively organized coacervation, yielding colloidal molecules with monodisperse size, morphological symmetry, and compositional heterogeneity. We investigate the coacervation of a mixture of two types of synthetic polymers and find that one of the polymers coacervates first and serves as a core droplet, on which the other polymer coacervates subsequently to form satellite droplets. The satellite droplets arrange themselves symmetrically around the core and solidify without losing the morphology. The number of satellites and their symmetry are modulable depending on the chemical affinity and the diameter of the droplets. This finding highlights the capability of coacervation as a non‐templated and non‐covalent pathway to form aspherical colloidal materials with structural and functional complexity.

## Graphical abstract

Synthesizing colloidal molecules through non‐template and non‐covalent coacervation processes is demonstrated. The sequential nucleation and growth of two types of synthetic polymers form compartmentalized droplet clusters, which grow by arranging the satellite droplets symmetrically and solidifying without losing the morphology. The resulting polymeric colloid molecules feature monodisperse size, morphological symmetry, and compositional heterogeneity.

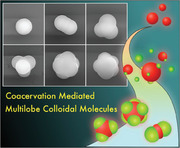

## 1 Introduction

Nano‐to‐micrometer scale colloids with less symmetry are of broad interest in the contemporary soft materials science to go beyond the conventional dense packing.^[^ ^1^, ^2^, ^3^, ^4^ ^]^ Clusters of colloids imitating the geometry and chemical heterogeneity of molecules offer a unique position and are termed colloidal molecules (CMs).^[^ ^5^, ^6^, ^7^ ^]^ As a macroscopic analogue of molecules, CMs can be harnessed with attractive or repulsive interactions at their periphery and can assemble into colloidal superstructures,^[^ ^8^, ^9^, ^10^ ^]^ whose structural complexities are exceedingly sophisticated in comparison to those composed of spherical counterparts.^[^ ^11^, ^12^, ^13^ ^]^ The superstructures are valuable as optically visible models to investigate how less symmetric colloidal objects organize to form a long‐range order^[^ ^14^, ^15^ ^]^ that are of particular interest for the photonics applications.^[^ ^16^, ^17^ ^]^

The synthesis of CMs with high uniformity, high production yield, and high reproducibility is a basis for expanding the research field. Because of their geometry and compositional heterogeneity, the synthesis generally involves multi‐step chemical routes.^[^ ^18^, ^19^, ^20^, ^21^, ^22^, ^23^ ^]^ Microphase separation of amphiphilic block copolymers (BCPs) in the emulsified droplets and in micelles has been proven as an effective method to obtain CMs.^[^ ^24^, ^25^, ^26^, ^27^ ^]^ However, the size of the resulting CMs is limited up to 100 nm due to the chain length of BCPs, though CMs with micrometer‐scale are preferred for photonics applications.

Coacervation can be an alternative synthetic route for overcoming this drawback. As is seen in living cells, coacervation is a biologically ubiquitous chemical process for organizing polymers into multicompartment droplets with a size of several micrometers.^[^ ^28^, ^29^ ^]^ The microdroplets exhibit various multicompartment structures, depending on the relative interfacial energy of the droplets in contact.^[^ ^30^, ^31^, ^32^ ^]^ Intriguingly, in the case of partial wetting interaction, the multicompartment droplets show multilobe geometries that are reminiscent of structures of simple molecules.^[^ ^33^, ^34^ ^]^ However, the available morphology, size, and symmetry of the multilobe structures are statistically distributed because the collision of the droplets is totally dominated by the kinetics. The solidification of the droplets without losing the structural order is another fundamental challenge for the applications, which is hindered by the concentration process that often induces the aggregation of the whole droplets.

In this context, we reported the bottom‐up synthesis of microspheres from π‐conjugated polymers using the solvent exchange method by vapor diffusion.^[^ ^35^, ^36^, ^37^ ^]^ Our previous studies using chiral conjugated polymers revealed that diffusion of a poor solvent vapor into a solution of polymer results in a liquid‐liquid phase separation (LLPS), forming polymer‐enriched droplets as the intermediate. Upon further diffusion of the solvent vapor, the spherical droplets are eventually solidified, forming the conjugated polymer microspheres.^[^ ^38^, ^39^ ^]^ These findings prompt us to investigate whether conjugated polymer droplets can form multicompartment structures.

In the present work, we report on a bottom‐up methodology for obtaining CMs with distinctively high size uniformity and morphological symmetry. Initially, two types of π‐conjugated polymers are dissolved in an organic solvent. Upon addition of a poor solvent by diffusing in a vapor phase, one of the polymers coacervates to form liquid droplets, on which the other polymer coacervates subsequently. Unlike conventional coacervation, the secondary droplets arrange themselves symmetrically around the core droplet to yield a molecular‐shaped cluster. The clusters are eventually solidified without losing the morphology. The strategy reported herein is a fully noncovalent, bottom‐up, and one‐pot process and is valuable for preparing CMs from various materials without using block copolymers. The phenomena found here are also informative for cultivating an insight into how liquid droplets develop their structural and functional complexity in the biological systems.

## 2 Results and Discussion

### 2.1 Formation of Molecular‐Shaped Microparticles from π‐Conjugated Polymer Blend

**Figure 1:**
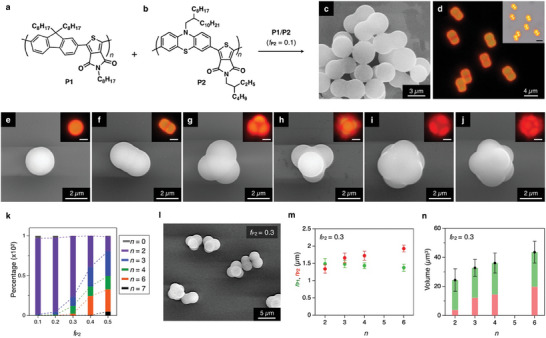
a, b) Molecular structures of π‐conjugated alternating copolymers **P1** (a) and **P2** (b). c) SEM micrographs of self‐assembled microstructures of **P1**/**P2** with *f* **_P2_** = 0.1. d) Fluorescence and optical (inset) micrographs of self‐assembled microstructures of **P1**/**P2** with *f* **_P2_** = 0.1. For FM, *λ*_ex_ = 400–440 nm. Scale bars (insets): 4 µm. e–j) SEM and FM (inset) micrographs of coassembled spherical (e, *n* = 0), dumbbell‐shaped (f, *n* = 2), triangular (g, *n* = 3), tetrahedral (h, *n* = 4), octahedral (i, *n* = 6), and pentagonal bipyramidal (j, *n* = 7) CMs. For FM, *λ*_ex_ = 400–440 nm. Scale bars (inset): 2 µm. k) Bar chart, showing the percentage of the multilobe CMs with *n* = 0 (grey), 2 (purple), 3 (blue), 4 (green), 6 (orange), and 7 (black) for different *f*_P2_. l) SEM micrograph of the resultant multilobe CMs at *f*_P2_ = 0.3. m) Plots of average *r* **_P1_** (green) and *r* **_P2_** (red) for the multilobe CMs versus *n* at *f*_P2_ = 0.3. n) Average volume of the multilobe CMs versus *n* at *f*_P2_ = 0.3. Red: central sphere, green: peripheral hemisphere.

Alternating copolymers **P1** (poly[(9,9‐dioctylfluorene‐2,7‐diyl)‐*alt*‐(5‐octylthieno[3,4‐c]pyrrole‐4,6‐dione‐1,3‐diyl)] with the number‐average molecular weight (*M*_n_) of 22.3 kg mol^−1^ (**P1**_22k_, **Figure** **1**a) and **P2** (poly[(10‐(2‐octyldodecyl)phenothiazine)‐*alt*‐(5‐[2‐ethylhexyl)‐thieno‐[3,4‐c]‐pyrrole‐4,6‐dione] with *M*_n_ of 15.0 kg mol^−1^ (Figure 1b) were synthesized according to the reported procedures.^[^ ^40^, ^41^ ^]^ It has been reported in our previous study that **P1** forms well‐defined microspheres.^[^ ^36^, ^37^ ^]^ As the counterpart for the coassembly, **P2** was selected because **P2** has resemble chemical structure with **P1**, expecting that **P2** will form microspheres under identical condition. Electronic photoabsorption spectra of CHCl_3_ solutions of **P1**_22k_ and **P2** show absorption maxima (λ_abs_) at 462 and 480 nm, respectively, while their photoluminescence (PL) spectra show PL maxima (*λ*_em_) at 487 and 608 nm (Figure S1, Supporting Information). The different PL colors can distinguish how two polymers assemble from the mixed state by fluorescence microscopy (FM) observations.

Self‐assembly of **P1**_22k_, and **P2**, and coassembly of their blends was conducted through the vapor diffusion (VD) method (Figure S2, Supporting Information).^[^ ^35^ ^]^ Typically, a 2‐mL glass vial containing CHCl_3_ solution of **P1**_22k_ (500 µL, [**P1**_22k_] = 0.5 mg mL^−1^) was put in a 20‐mL glass vial containing 2.5 mL of MeOH. The outer vial was capped and stood at 25 °C to allow MeOH vapor to diffuse into the CHCl_3_ solution of the polymers. After 24 h, a yellowish precipitate resulted at the bottom of the small vial. Scanning electron microscopy (SEM) micrograph shows well‐defined microspheres of **P1**_22k_ with an average diameter (*d*_av_) of 2.4 µm and the standard deviation (*σ*) of 0.19 µm (Figure S3a, Supporting Information). In a similar protocol, **P2** forms microspheres with *d*_av_ and *σ* of 3.0 and 0.36 µm, respectively (Figure S3b, Supporting Information). The microspheres of **P1**_22k_ and **P2** show green and red fluorescence, respectively, in the FM images (Figures S3c,d, Supporting Information).

When the VD process was conducted with a solution of a blend of **P1**_22k_ and **P2** with the weight fraction of **P2** (*f* **_P2_**) of 0.1, dumbbell‐shaped microparticles were precipitated (Figure 1c). FM images of the dumbbell‐shaped microstructures display orange‐color PL on both sides, while the middle part displays red‐color PL (Figure 1d). These images indicate that **P1**_22k_ and **P2** are not miscible with one another but are compartmentalized to form the dumbbell structure, where the central sphere composed of **P2** is sandwiched by the two spheres composed of **P1**_22k_. The PL color of both sides of the dumbbell is orange, not green, because a small portion of **P2** is mixed in the sphere of **P1**_22k_. The micrometer‐scale‐PL (µ‐PL) measurements of the dumbbell‐shaped structures revealed that the PL spectrum collected from the side sphere, even *f* **_P2_** = 0.1, is almost identical to that of **P2** dissolved in CHCl_3_ (Figure S4, Supporting Information). This result clearly indicates that Förster resonance energy transfer (FRET) occurs efficiently from **P1**_22k_ to slightly mixed **P2** in the side sphere because of the large overlap of the PL band of **P1**_22k_ with the absorption band of **P2** (Figure S1, Supporting Information).^[^ ^42^, ^43^ ^]^

To investigate the formation of the compartmentalized colloids, coassembly of **P1**_22k_/**P2** with various *f* **_P2_** was conducted, and the morphology and population of the resultant precipitates were confirmed from ≈300 particles for each *f* **_P2_** condition. The morphology is categorized based on the number of the peripheral hemispheres (*n*). For *f* **_P2_** = 0.1, more than 95% of the particles form the dumbbell‐shaped structures (*n* = 2, *D*_∞h_), and the rest is the simple sphere (*n* = 0, Figure 1e,f,k). The dumbbell‐shaped particles are the major product for *f* **_P2_** = 0.2 and 0.3, and a portion of the particles forms a triangular (*n* = 3, *S*_3_), tetrahedral (*n* = 4, *T*_d_), and octahedral (*n* = 6, *O*_h_) symmetric structures (Figure 1g–i,k,l). The pentagonal bipyramidal structure (*n* = 7, *D*_5h_) was observed as the largest *n* for *f* **_P2_** of 0.5 (Figure 1j). The discrete multilobe colloids are reminiscent of the space‐filling model of molecules and are literally referred to as CMs.^[^ ^5^, ^6^ ^]^ On the other hand, as *f*_**P2**_ increases more than 0.5, the yield of CMs decreases. When *f*_**P2**_ reaches 0.8, most of the precipitates are agglomerated, and the symmetry of CMs is hardly discernible (Figure S5, Supporting Information).

The average radii of the peripheral spheres of **P1**_22k_ (*r* **_P1_**) and the central sphere of **P2** (*r* **_P2_**) at *f* **_P2_** = 0.3 are plotted in Figure 1m. The *r* **_P1_** values are almost identical with respect to *n* (1.4–1.5 µm), while the *r* **_P2_** values increase from 1.3 to 2.0 µm as *n* increases from 2 to 6. We further evaluate the average volume (*V*_av_) of each compartment with the approximation that the CMs are the combination of the truncated central sphere of **P2** and hemispheres of **P1**_22k_ (Table S1, Supporting Information).^[^ ^24^ ^]^ The *V*_av_ values for *n* = 2, 3, 4, and 6 are 24.3, 31.4, 36.5, and 41.7 µm^3^, respectively (Figure 1n). The bar chart shows a clear dependence of *V*_av_ on *n*. Considering the radius and volume fraction of the peripheral and central spheres, it is concluded that *n* increases as the size of the central sphere increases, and the size of the central (**P2**) sphere determines the symmetry of the CMs. We further conduct statistical analyses of the size distribution of the colloids at different *f* **_P2_** (Figure S6, Supporting Information). As a result, the standard deviation (*σ*) and the coefficient of variation (*C*_V_) values of the colloids at a given *f* **_P2_** are less than 0.32 µm and 5–11%, respectively. These values are sufficient to be considered as monodisperse colloids.^[^ ^44^ ^]^

### 2.2 Formation Mechanism of Polymeric CMs in Vapor Diffusion Process

**Figure 2:**
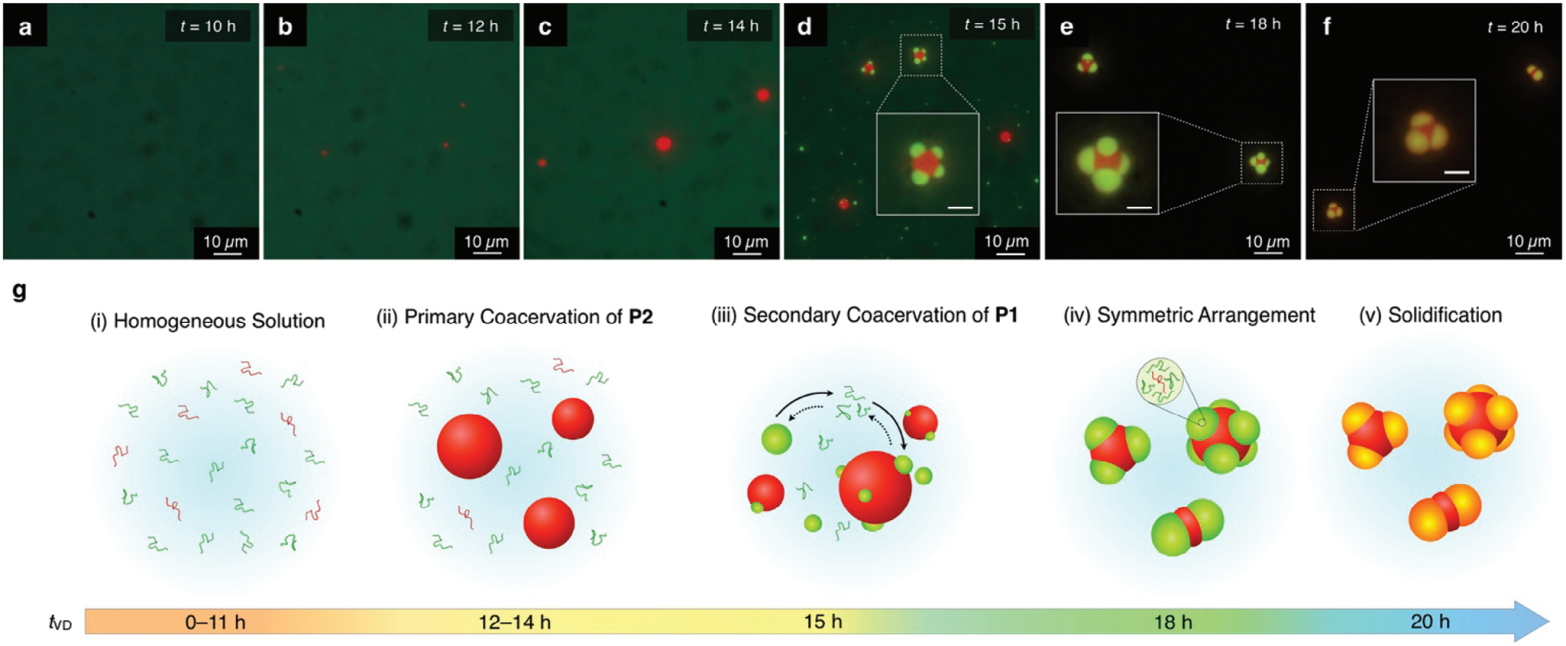
a–f) Fluorescent micrographs of coassembly of **P1**_22k_ /**P2** with *f* **_P2_** = 0.4 upon diffusion of MeOH vapor at *t*_VD_ = 10 (a), 12 (b), 14 (c), 15 (d), 18 (e), and 20 h (f). *λ*_ex_ = 400–440 nm. Scale bars of the insets of d–f: 3 µm. g) Schematic representations of the plausible formation mechanism of coassembled π‐conjugated polymer CMs from isotropic solution (i), primary LLPS of **P2** (ii), secondary LLPS of **P1**_22k_ both on the **P2** droplets and in the solution (iii) and rearrangement into symmetrical positions under isotropic pressure (iv) upon condensation.

To gain insight into the formation process of the π‐conjugated polymer CMs, we took time‐course microscopic images of the formation of CMs. An aliquot of the solution of the polymer blend with *f* **_P2_** = 0.4 was collected from the vial during the VD process and subjected to FM observation (Figures S7 and S8, Supporting Information). Until 11 h of the vapor diffusion (*t*_VD_ = 11 h), the blend solution was homogeneous without any precipitates (**Figure** **2**a). At *t*_VD_ = 12 h, spherical micro‐colloids of **P2** with red‐colored fluorescence appeared in the solution (Figure 2b), and the colloids grew larger at *t*_VD_ = 14 h (Figure 2c). At *t*_VD_ = 15 h, multiple small colloids with green fluorescence appeared both on the primary **P2** colloids and in the solution (Figure 2d). Because the colloids fuse with each other upon their collision, the red‐ and green‐fluorescent colloids at *t*_VD_ 12–15 h are the liquid droplets of enriched **P2** and **P1**_22k_, respectively, via LLPS (Movies S1 and S2, Supporting Information). As the VD process further proceeded (*t*_VD_ = 18 h), the **P1**_22k_ droplets in contact with **P2** droplets grew larger while maintaining the compartments and arranged at the polyhedral vertex positions of the multilobe structure. Simultaneously, the small **P1**_22k_ droplets in the solution disappeared (Figure 2e). Finally, at *t*_VD_ = 20 h, the compartmentalized droplets were solidified, and the color of the peripheral **P1**_22k_ compartment turned from green to orange, attributed to FRET from **P1**_22k_ to **P2** by the solidification (Figure 2f).

Based on the time‐course change of the morphology, we propose a plausible mechanism for the spontaneous formation of CMs (Figure 2g). Because of the poor solubility of **P2** to MeOH (Figure S9, Supporting Information), the addition of MeOH vapor to the solution of the polymers causes the primary coacervation of **P2** (Figure 2g (ii)). Further addition of MeOH vapor results in the secondary coacervation of **P1**_22k_ both in the solution and at the interface of the droplet of **P2**. The **P1**_22k_ droplet attached to the **P2** droplet is more energetically favorable than those solely appear in the solution, resulting in the Ostwald ripening of the **P1**_22k_ droplets and disappearance of the **P1**_22k_ droplets in the solution (Figure 2g (iii)). Because the **P1**_22k_ droplets are not fixed on the surface of the **P2** droplets, symmetric arrangement of the **P1**_22k_ droplets at the polyhedral vertex positions occurs so that the distortion stress of the **P2** droplet is minimized (Figure 2g (iv)).

Because the diffusion of MeOH vapor into the polymer blend solution is sufficiently slow, the stepwise coacervation proceeds under the quasi‐equilibrium control. The structure of the droplets at each equilibrium condition is described based on the relative interfacial tensions *γ*_1_, *γ*_2_, and *γ*_12_, which respectively represent the interfacial tensions between the **P1**_22k_ droplet and the dilute phase (surrounding solution), the **P2** droplet and the dilute phase, and the **P1**_22k_ droplet and the **P2** droplet. According to the formation of the non‐spherical compartmentalized droplets, the relative interfacial tensions must be in a range of partial wetting, which appears as a case other than engulfing (*γ*_1_ > *γ*_2_ + *γ*_2_ or *γ*_2_ > *γ*_1_ + *γ*_12_) or separation (*γ*_12_ > *γ*_1_+ *γ*_2_) (Figure S10, Supporting Information).^[^ ^28^, ^30^ ^]^ Throughout the stepwise coacervations, the central **P2** droplets are dented by the hemispherical **P1**_22k_ droplets (Figure 2d–f). This result indicates that *γ*_1_ is slightly higher than *γ*_2_, and *γ*_2_ is comparable with *γ*_12_.^[^ ^45^, ^46^ ^]^ This interfacial valance enables the fabrication of molecular‐shaped polymeric colloids through a spontaneous self‐assembly process.

### 2.3 Effect of *M*_n_ on the Morphology of the Colloidal Molecules

**Figure 3:**
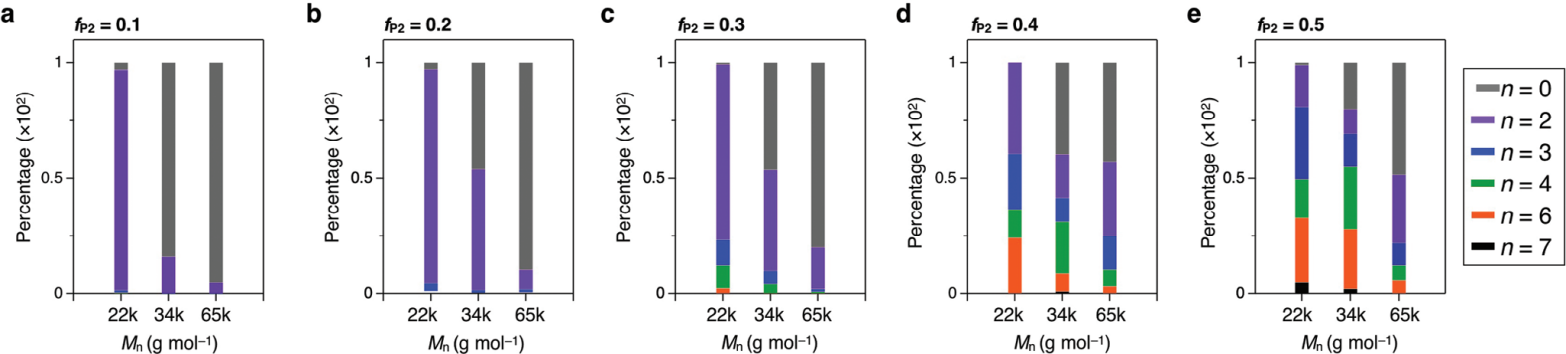
a–e) Bar charts, showing the percentage of the multilobe CMs with *n* = 0 (grey), 2 (purple), 3 (blue), 4 (green), 6 (orange) and 7 (black) for different *f* **_P2_** by coassembly of **P1**_22k_/**P2**, **P1**_34k_/**P2**, and **P1**_65k_/**P2**.

We previously reported that the nucleation timing and growth rate of **P1** are dependent on *M*_n_: The larger *M*_n_ leads to the earlier nucleation timing and faster growth rate.^[^ ^36^, ^37^ ^]^ This knowledge motivates us to investigate how the different *M*_n_ influences the coacervation process and resultant microstructures. For this purpose, we synthesized **P1** with larger *M*_n_, 33.6 and 64.5 kg mol^−1^, which are abbreviated as **P1**_34k_ and **P1**_65k_, respectively. By the similar VD process, **P1**_34k_ and **P1**_65k_ of each form well‐defined microspheres with *d*_av_ of 2.3 µm (*σ* = 0.35 µm) and 2.0 µm (*σ* = 0.34 µm), respectively (Figure S11, Supporting Information). Coassembly of **P1**_34k_/**P2** and **P1**_65k_/**P2** were conducted with *f* **_P2_** ranging from 0.1 to 0.5. **Figure** **3**a–e shows the bar chart on the percentage of the resultant colloidal morphologies at *f* **_P2_** = 0.1–0.5. The significant trend is that, as increasing *M*_n_ of **P1**, the population of the CMs (*n* ≥ 2) decreases relative to that of isotropic spherical particles (*n* = 0). For example, at *f* **_P2_** = 0.1, **P1**_34k_/**P2** and **P1**_65k_/**P2** dominantly form spherical microparticles (*n* = 0) with percentages of 84% and 95%, respectively. The isotropic spheres show orange fluorescence, indicating that the spheres are composed dominantly of **P1** with a small amount of **P2** (Figure S12, Supporting Information). As *f* **_P2_** increases, the percentages of the isotropic spheres decrease but remain at 20% and 50% for *f* **_P2_** = 0.5.

We conducted the time‐course observations of the coassembly process of **P1**_65k_/**P2** at *f* **_P2_** = 0.1. In contrast to that of **P1**_22k_/**P2**, **P1**_65k_ and **P2** coacervated almost simultaneously at *t*_VD_ = 12 h and solidified at *t*_VD_ = 18 h, forming microspheres with orange fluorescence (Figure S13, Supporting Information). This result indicates that simultaneous coacervation of the polymer blend solution results in the formation of droplets that contain both polymers, not forming phase‐separated multilobe CMs. This is consistent with our previous studies, where polymer blend microspheres were obtained through precipitation of a polymer blend induced by the vapor‐phase diffusion of a poor solvent.^[^ ^36^, ^47^ ^]^

Considering the results of the coassembly of **P1** with different molecular weights and *f* **_P2_**, selective formation of CMs for *n* ≥ 3 seems a great challenge. The formation of CMs with *n* = 2 (dumbbell‐shape) is almost uniform at *f*_P2_ = 0.1, at which the radius of the core is less than 1.5 µm. However, as shown in Figure 1m, the radii of the core spheres for *f* **_P2_** = 0.3 are in the range of 1.2–2.0 µm. The range of *σ* for the average radii of the core is overlapped with one another for *n* ≥ 3, resulting in the formation of a mixture of CMs with different *n*.

Since the radius of the self‐assembled polymer microspheres depends on the initial concentration of polymers,^[^ ^38^ ^]^ we performed the polymer coassembly at *f* **_P2_** = 0.3 with varying initial concentrations (*c*) from 0.05 to 2.5 mg mL^−1^. As a result, the abundance ratio of the resultant symmetric structures was nearly the same in the range of 0.05–1.0 mg mL^−1^, but the selective formation of a single sort of CMs has not been achieved (Figure S14, Supporting Information).

Note that, when the volume of the solvent was increased by 4, 10, and 20 times, the resultant CMs were not well isolated but merged (Figure S15, Supporting Information). These results indicate that the formation of CMs is sensitive to the batch scale of the assembly as well as the concentration, molecular weight, and mixing ratio of the polymers.

### 2.4 Coacervation for Combinations of Various Fluorescent Polymers

**Figure 4:**
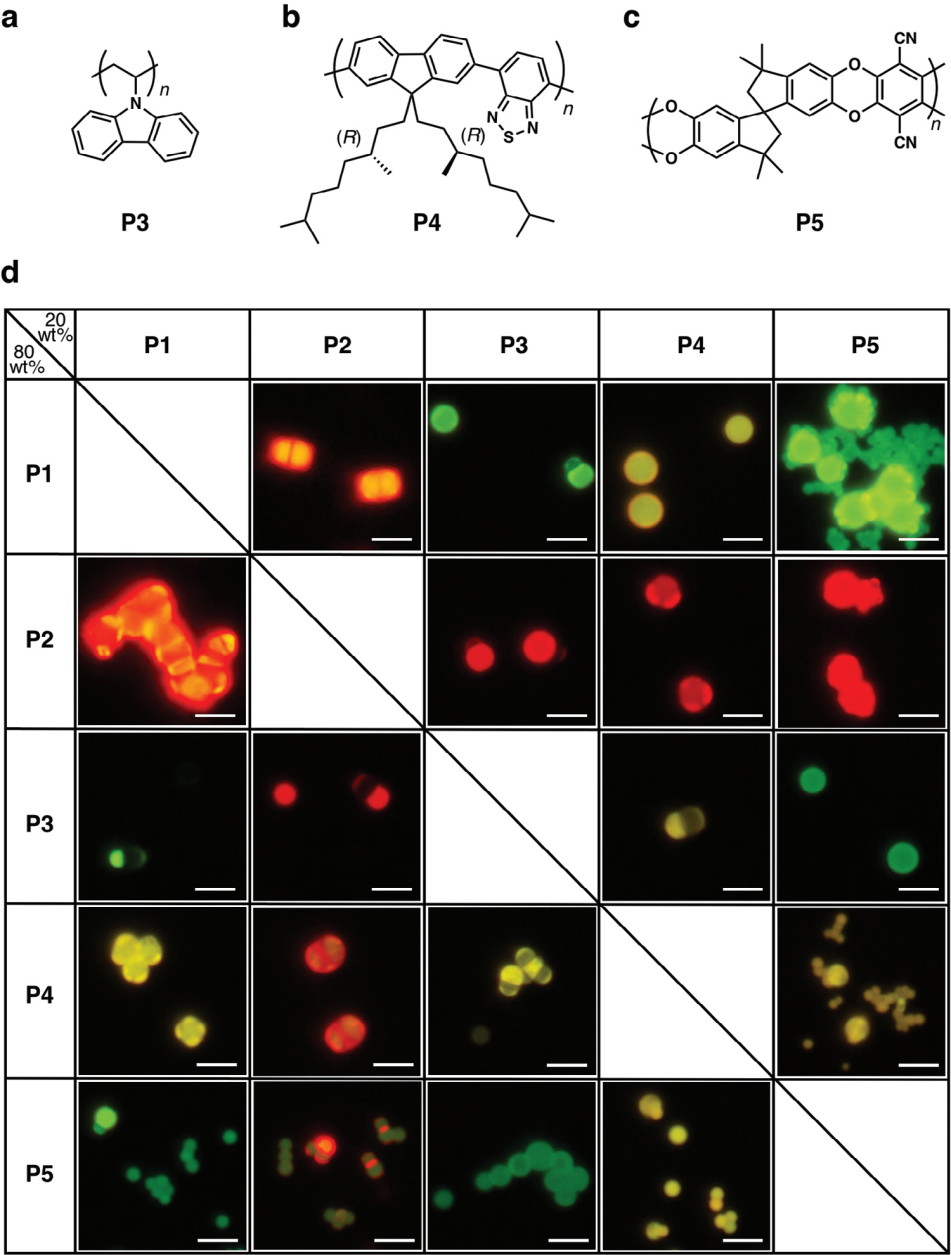
a–c) Molecular structures of **P3** (a), **P4** (b), and **P5** (c). d) Tabular summary of FM images of the resultant CMs prepared from binary polymer blends of **P1**_22k_, **P2**, **P3**, **P4**, **P5** with the weight ratio of 8/2. Scale bars: 5 µm.

To prove the concept for the formation of π‐conjugated polymer CMs, we further conducted the coassembly of binary polymer blends systematically mixed from five kinds of polymers that are known to form microspheres via the VD method. Namely, **P1**_22k_, **P2**, **P3** (poly(9‐vinylcarbazole)), **P4** (poly(9,9‐bis((*R*)‐3,7‐dimethyloctyl)‐2,7‐fluorene‐*alt*‐benzothiadiazole)),^[^ ^38^, ^39^ ^]^ and **P5** (polymer of intrinsic microporosity, **PIM‐1**, **Figure** **4**a–c and Figure S16, Supporting Information).^[^ ^48^ ^]^ For each blend combination, coassembly was performed with the weight ratio of one polymer to the other of 2/8.

The FM and OM images of the resultant microstructures are tabularly summarized in Figure 4d and Figure S17 (Supporting Information), respectively. For instance, the coassembly of **P2**/**P4** tends to form multilobe CMs regardless of the weight ratio, whereas the coassembly of **P1**_22k_/**P4** forms either multilobe CMs or blend microspheres depending on the weight ratio. The combinations of **P3** with **P1**_22k_, **P2**, and **P4** tend to form snowman‐shaped CMs (*n* = 1) as well as microspheres. According to the colorless FM images from the **P3** compartments, **P3** is virtually immiscible with these polymers and occasionally forms CMs with a single lobe to minimize the contact surface area (Figure S18, Supporting Information). In contrast, the combination of **P3** and **P5** is highly miscible with each other, yielding blend microspheres with green fluorescence. In the case of the combination of **P5** with **P1**_22k_ and **P2**, multilobe colloids covered with tiny peripheral particles are formed in case **P5** is the minor fraction, while microspheres and a few CMs are formed when **P5** is the major fraction.

### 2.5 Parameters for Determining the Colloidal Structure

**Figure 5:**
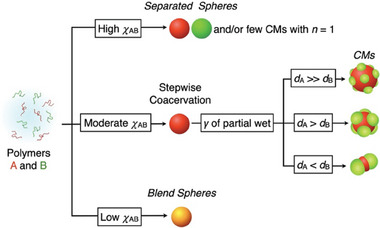
Schematic diagram of the parameters for determining the final colloidal structure. *χ*_AB_: Flory‐Huggins interaction parameter between polymers A and B, *γ*: relative interfacial tension, *d*_A_, *d*_B_: diameters of the droplets of polymer A and B.

We elucidate the mechanism of the clustering based on the results obtained in the current and previous studies,^[^ ^37^ ^]^ ending up with a concept as summarized in **Figure** **5**. Therein, the chemical affinity (miscibility) between the two polymers is the primary parameter determining the outcome of clustering, which has been discussed traditionally in the solid state based on the Flory‐Huggins interaction parameter (*χ*).^[^ ^49^, ^50^, ^51^, ^52^ ^]^ Given that the polymers are labeled as A and B, and *χ*_A/B_ is significantly low, the two polymers A and B mix together completely at the molecular scale and form blend microspheres (Figure 4d and Figure S17, Supporting Information, **P3**/**P5**).^[^ ^36^ ^]^ In case *χ*_A/B_ is high enough, the polymers are thoroughly immiscible with each other and form coacervates separately (Figure 4d and Figure S17, Supporting Information, **P1**/**P3** and **P2**/**P3**).

Intriguing is a pair of polymers with moderate *χ*_A/B_. These polymers are basically immiscible and coacervate separately in an analogy to the highly immiscible pair. Meanwhile, when the droplets of A and B are slightly affinitive with each other, it allows the nucleation and adhesion of droplet of polymer B, for instance, at the surface of the droplet of polymer A as experimentally visualized in Figure 2, though they are immiscible. The secondary nucleation at the surface is facilitated progressively when the primary coacervate droplets of polymer A grow much faster than polymer B due to the enlarged surface of the core droplets. The interval between the coacervation of two polymers should be large enough to enrich the secondary nucleation, as shown in the discussion in Figure 3. The vapor diffusion method is favorable for extending the interval between the coacervation of the two polymers and for allowing the core droplets to grow sufficiently owing to the significantly slow and calm change in solubility in comparison to the other conventional methodology.

The geometry of the clusters is dominated by the relative interfacial tension *γ* between polymers A and B. It is well known that the geometry of manually clustered immiscible droplets becomes molecular mimetic when *γ* is moderate, which is categorized as partial wetting. Otherwise, the droplets end up with separation or engulfment (Figure S10, Supporting Information), meaning that CMs in interest are provided from a pair of polymers with moderate *χ*_A/B_ and moderate *γ*. We do not think it a coincidence that the pairs of polymers **P1**/**P2**, **P1/P4**, and **P2/P4** satisfy this strict requisite of *γ* and *χ*_A/B_ simultaneously because both *χ*_A/B_ and *γ* have their origin in the affinity of the polymers. Finally, the number of satellites surrounding the core is determined by the diameter of the core, as deduced from the results in Figure 1m,n.

## 3 Conclusion

We present a fully noncovalent and one‐pot spontaneous formation of molecular‐shaped microparticles through stepwise coacervation of the binary polymer blend solution. Detailed time‐course microscopic images of the polymer blend solution reveal that the coassembly of **P1**_22k_ and **P2** self‐organizes to form non‐spherical compartmentalized droplets as intermediates via coacervation of **P2** followed by that of **P1**_22k_. Due to the smaller interfacial penalty, droplets of **P1**_22k_ in contact with that of **P2** initiatively grow throughout the successive intrusion of MeOH vapor. In the meantime, the peripheral droplets are arranged at the polyhedral vertex positions to minimize the distortion stress posed on the central one. The fluidic clusters eventually solidify without losing their morphology. Systematic analysis of the coassembly of **P1**_22k_/**P2**, **P1**_34k_/**P2**, and **P1**_65k_/**P2** indicates that the *M*_n_ of the constituent polymers largely affects the formation of the CMs. Results on the coacervations of the binary polymer mixtures using five kinds of *π*‐conjugated polymers clarify the parameters for determining the formation and shapes of the final colloidal structure. This study highlights the capability of coacervation as a non‐template and non‐covalent pathway to organize intricate colloidal materials, which broadens the field of supramolecular colloidal materials with structural and functional complexity and shows how the physical chemistry of coacervation is versatile in the synthetic and biological systems.

## Conflict of Interest

The authors declare no conflict of interest.

## Supporting information



Supporting Information

Supplemental Movie 1

Supplemental Movie 2

## Data Availability

The data that support the findings of this study are available from the corresponding author upon reasonable request.
