# Supplementary material for: Spontaneous Formation of π‐Conjugated Polymeric Colloidal Molecules Through Stepwise Coacervation and Symmetric Compartmentalization
Source: Small. 2024 Oct 10;21(5):2404934. doi: 10.1002/smll.202404934 (PMC11798348; doi:10.1002/smll.202404934)
Supplement: Supplementary file 1 — Supporting Information [file SMLL-21-2404934-s001.pdf]

NANO · MICRO
small

Supporting Information

for *Small*, DOI 10.1002/smll.202404934

Spontaneous Formation of π-Conjugated Polymeric Colloidal Molecules Through Stepwise Coacervation and Symmetric Compartmentalization

Osamu Oki, Shun-ichiro Noguchi, Sota Nakayama, Hiroshi Yamagishi, Junpei Kuwabara, Takaki Kanbara and Yohei Yamamoto**

Supporting Information

Spontaneous Formation of π-Conjugated Polymeric Colloidal Molecules through Stepwise Coacervation and Symmetric Compartmentalization

Osamu Oki,†*[a,b] Shun-ichiro Noguchi, †[a] Sota Nakayama,[a] Hiroshi Yamagishi,[a] Junpei Kuwabara,[a] Takaki Kanbara,[a] Yohei Yamamoto*[a]

a Department of Materials Science, Institute of Pure and Applied Sciences, and Tsukuba Research Center for Energy Materials Science (TREMS), University of Tsukuba, 1-1-1 Tennodai, Tsukuba, Ibaraki 305-8573, Japan

b Institute for Complex Molecular Systems and Laboratory of Macromolecular and Organic Chemistry, Eindhoven University of Technology, 5600 MB Eindhoven

† These authors contributed equally to this work.

*e-mail: s-oki@ims.tsukuba.ac.jp, yamamoto@ims.tsukuba.ac.jp

1. General

Materials and Methods

All the chemicals and reagents were purchased from Sigma-Aldrich, TCI, and Merck. Solvents were obtained from Fujifilm Wako Chemicals. Unless otherwise noted, all reagents and solvents were used as received. Conjugated alternating copolymer, poly[(9,9-dioctylfluorene-2,7-diyl)-*alt*-(5-octylthieno[3,4-c]pyrrole-4,6-dione-1,3-diyl)], **P1**, (Figure 1a, M_n = 22.3 kg mol^{-1} with polydispersity index (PDI) = 1.8, M_n = 33.6 kg mol^{-1} with PDI = 2.0, M_n = 64.5 kg mol^{-1} with PDI = 2.1, M_n = 31.0 kg mol^{-1} with PDI = 1.9, only used in Figure S15),[S1] poly[(10-(2-octyldodecyl)phenothiazine)-*alt*-(5-[2-ethylhexyl)-thieno-[3,4-c]-pyrrole-4,6-dione], **P2**, (Figure 1b, M_n = 15.0 kg mol^{-1}, PDI = 1.8),[S2] poly(9,9-bis((*R*)-3,7-dimethyloctyl)-2,7-fluorene-alt-benzothiadiazole), **P4**, (Figure 4b, M_n = 9.5 kg mol^{-1} with PDI = 2.2),[S3] polymer of intrinsic microporosity, **P5**, (Figure 4c, M_n = 40.8 kg mol^{-1} with PDI = 2.1) [S4] were synthesized according to the reported procedures. Poly(9,9-dioctylfluorene-*alt*-benzothiadiazole), **P3**, (Figure 4a, M_n = 25~50 kg mol^{-1}) was purchased from Sigma–Aldrich. Electronic photoabsorption spectra were recorded on a JASCO V-630 spectrophotometer. Steady-state PL spectra were measured on a JASCO FP-8300 spectrofluorometer. SEM was performed on a Hitachi model S-3700N SEM operating at 20 kV. Optical, fluorescent, and polarized optical microscopic (OM, FM, and POM, respectively) observations were carried out using an Olympus model BX53 Upright Microscope.

Calculation of coefficient of variation

Coefficient of variation (C_V) was calculated by the equation below.

$$C_V = (\sigma/\text{average size}) \times 100$$

In general, C_V values less than 10% is considered as good monodispersity.

Time-course observations of a coassembly of P1 and P2

$CHCl_3$ solution of the polymer blend solution of **P1** and **P2** with an optional $f_{\mathbf{P2}}$ (0.5 mg mL^{-1}, 500 µL) was placed in a 20 mL vial containing 2.5 mL of MeOH as a nonsolvent. The out-side vial was sealed with a lid with a syringe and then allowed to stand for their co-assembly. At each target time, 1.0 µL of the solution (or the suspension) was dropped on one cover glass and gently covered with the other cover glass to prepare a specimen. Self-assembled structures in the specimen were immediately characterized by the fluorescent microscopy (Figure S7).

2. Flory-Huggins equation for polymer blend solution system

From thermodynamic Flory-Huggins model, Gibbs free energy of mixing (ΔG_{mix}) for a polymer blend solution system (polymer A, polymer B, and a solvent) can be described as below.[S5]

$$\frac{\Delta G_{mix}}{RT} = \frac{\varphi_S}{r_S}\ln\varphi_S + \frac{\varphi_A}{r_A}\ln\varphi_A + \frac{\varphi_B}{r_B}\ln\varphi_B + \chi_{SA}\varphi_S\varphi_A + \chi_{SB}\varphi_S\varphi_B + \chi_{AB}\varphi_A\varphi_B$$

where φ is the volume fraction, r is the number of segments per molecule, χ is the Flory-Huggins interaction parameter between the components, R is the gas constant, and T is the absolute temperature.

Solvent-polymer χ parameter is known to be described by the solubility parameters. For instance, χ_{SA} can be written as

$$\chi_{SA} = \frac{V_S}{RT}(\delta_S - \delta_A)^2 + 0.34$$

where V_S is the molar volume of the solvent, δ_S and δ_A are the solubility parameter of the solvent and polymer A. For example, δ_{CHCl3} and δ_{MeOH} are known to 9.4 and 14.5, $MPa^{0.5}$ respectively.[S5] Furthermore, the concept of solubility parameters is often extended to polymers. For instance, δ_{P3} is reported as 9.97 $MPa^{0.5}$. [S6] Although δ for other polymers used in this study are not known, δ of typical aromatic polymers such as polyfluorene (PFO) and poly(9,9-dioctylfluorene-*alt*-benzothiadiazole) (PFBT) are known to a range from 9 to 10 $MPa^{0.5}$.[S7,S8]

3. Supporting Figures

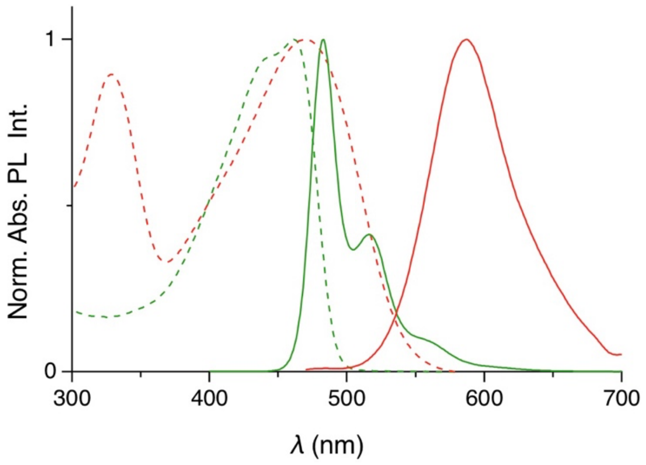

Figure S1. Photoabsorption (dashed line) and PL (solid line) spectra of **P1**$_{22k}$ (green) and **P2** (red) in $CHCl_3$.

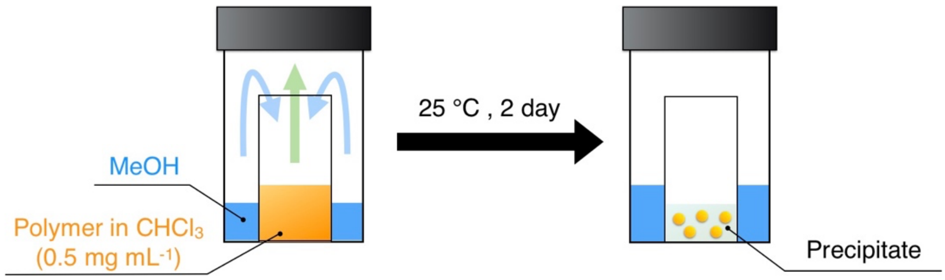

Figure S2. Schematic representation of a typical preparation procedure of polymer microspheres by vapor diffusion method.

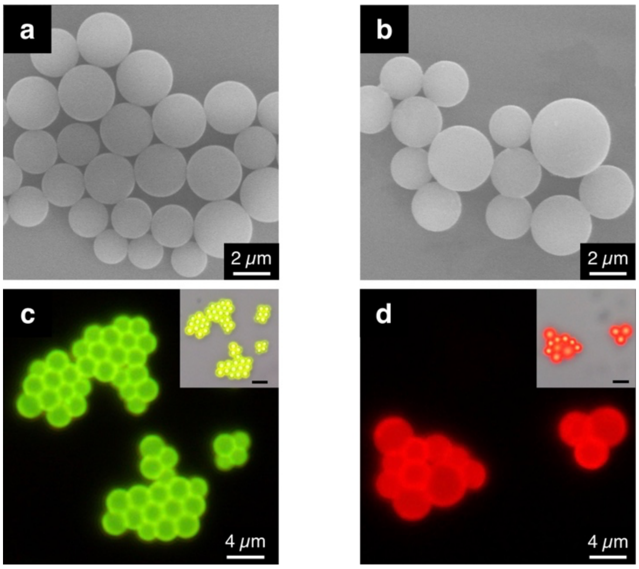

Figure S3. (a, b) SEM micrographs of self-assembled microspheres of **P1**$_{22k}$ (a) and **P2** (b). (c, d) FM and optical (inset) micrographs of self-assembled microspheres of **P1**$_{22k}$ (c) and **P2** (d). For FM, λ_{ex} = 400–440 nm. Scale bar (inset): 4μm.

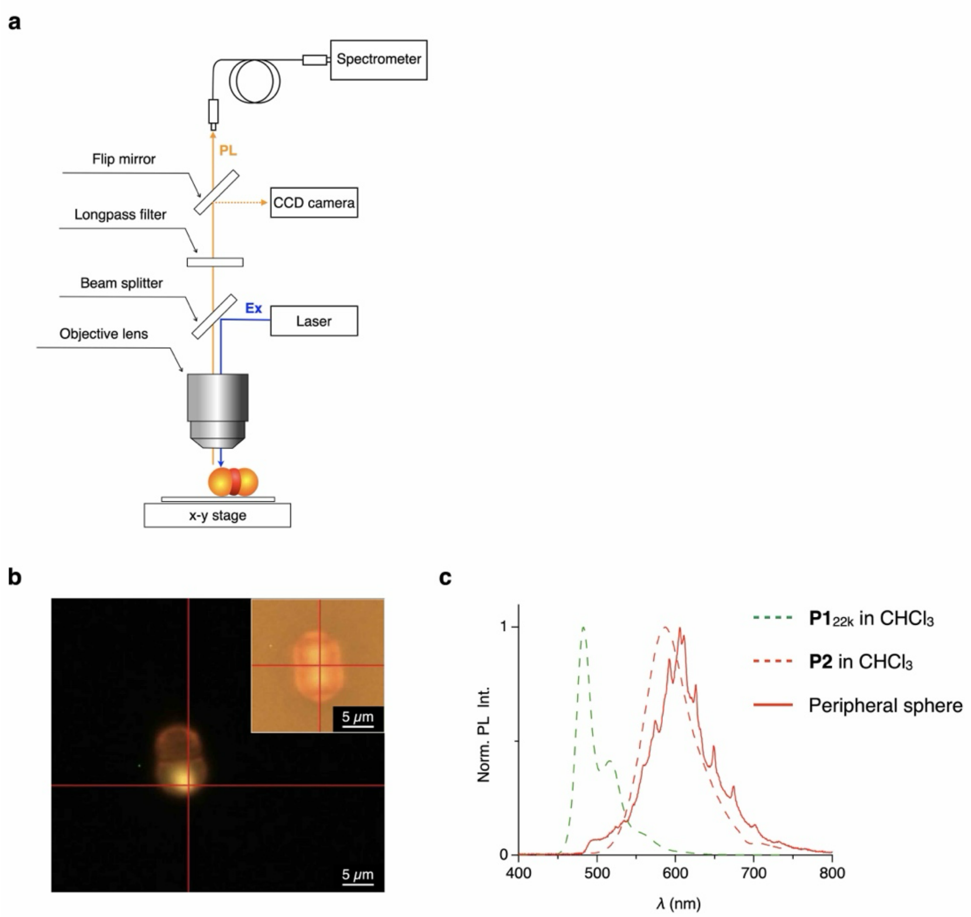

Figure S4. (a) Schematic representation of μ-PL setup. (b) Fluorescence and optical (inset) micrographs of a dumbbell-shaped CM. The side microsphere was selectively excited by a focused laser beam with 405 nm. (c) PL spectra collected from the side microsphere (red, solid curve), **P1** dissolved in $CHCl_3$ (green, broken curve), and **P2** dissolved in $CHCl_3$ (red, broken curve). The periodic PL lines overlapping on a broad PL band observed from the side microsphere is originated from the whispering gallery mode (WGM) optical resonance.

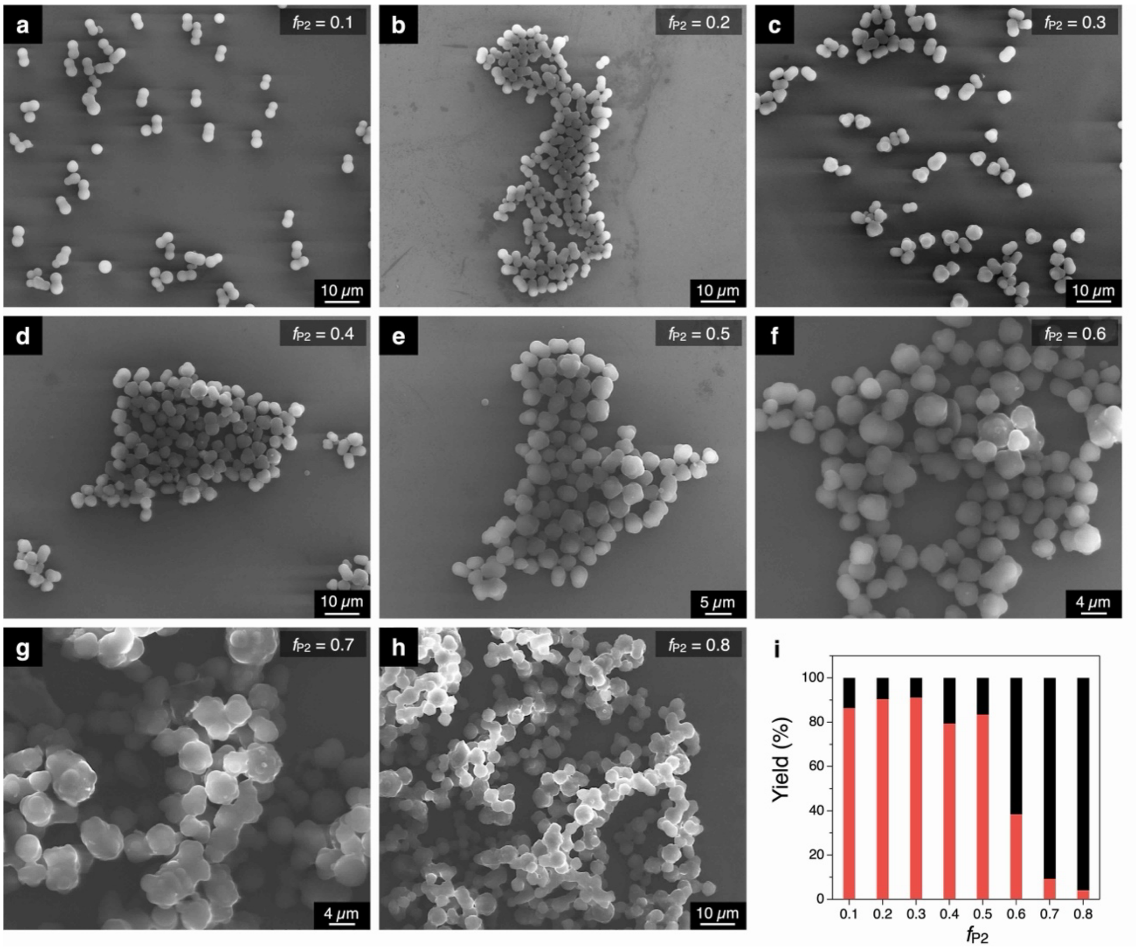

Figure S5. (a-h) SEM micrographs of coassembly of **P1**$_{22k}$/**P2** with $f_{\mathbf{P2}}$ = 0.1 (a), 0.2 (b), 0.3 (c), 0.4 (d), 0.5 (e), 0.6 (f), 0.7 (g), 0.8 (h). (i) Yield of the formation of well-defined CMs (red) and discernible particles (black) with respect to different $f_{\mathbf{P2}}$.

Table S1. Volume equations for coassembled colloids with different *n*.

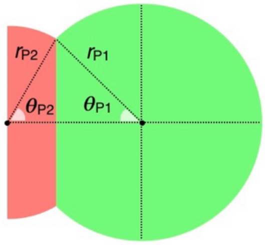

$$V_{n=2} = V_{P1} + V_{P2} = 2\left[\frac{\pi}{3} r_{P1}^3 (2 + 3\cos\theta_{P1} - 3\cos^3\theta_{P1}) + \frac{\pi}{3} r_{P2}^3 (3\cos\theta_{P2} - \cos^3\theta_{P2})\right]$$

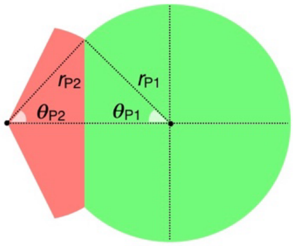

$$V_{n=3} = V_{P1} + V_{P2} = 3\left[\frac{\pi}{3} r_{P1}^3 (2 + 3\cos\theta_{P1} - 3\cos^3\theta_{P1}) + \frac{\pi}{3} r_{P2}^3 \left(-\frac{2}{3} + 3\cos\theta_{P2} - \cos^3\theta_{P2}\right)\right]$$

$$V_{n=4} = V_{P1} + V_{P2} = 4\left[\frac{\pi}{3} r_{P1}^3 (2 + 3\cos\theta_{P1} - 3\cos^3\theta_{P1}) + \frac{\pi}{3} r_{P2}^3 (-1 + 3\cos\theta_{P2} - \cos^3\theta_{P2})\right]$$

$$V_{n=6} = V_{P1} + V_{P2} = 6\left[\frac{\pi}{3} r_{P1}^3 (2 + 3\cos\theta_{P1} - 3\cos^3\theta_{P1}) + \frac{\pi}{3} r_{P2}^3 \left(-\frac{4}{3} + 3\cos\theta_{P2} - \cos^3\theta_{P2}\right)\right]$$

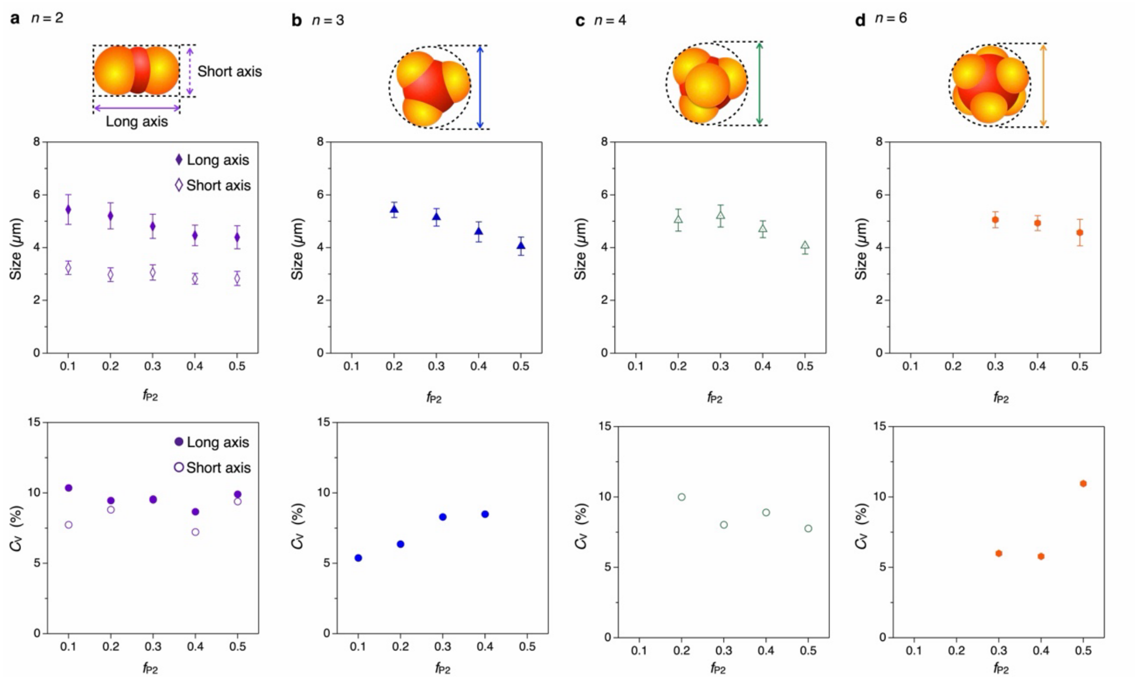

Figure S6. Plots of the size distributions and C_V of the multilobe CMs with $n = 2$ (a), 3 (b), 4 (c), and 6 (d) with dependent $f_{\mathbf{P2}}$.

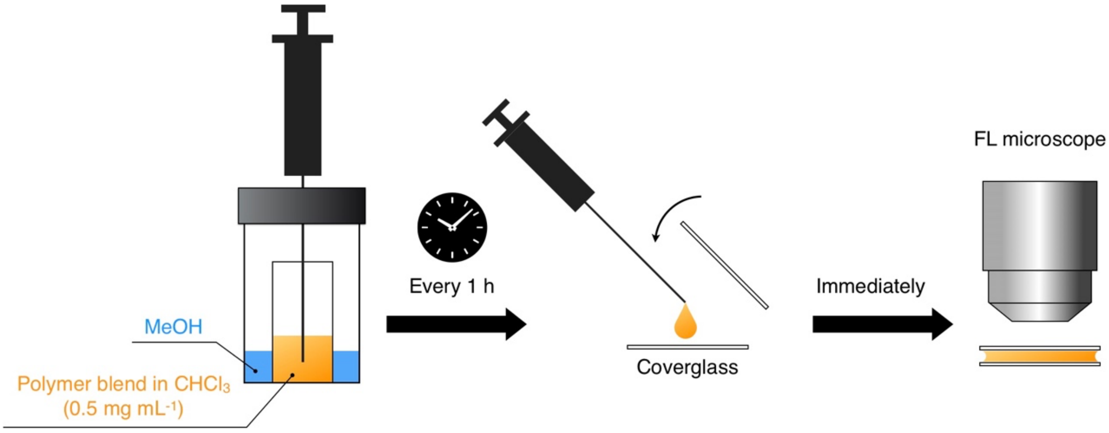

Figure S7. Schematic representation of a procedure of time-course FL microscopic observation of the coassembly.

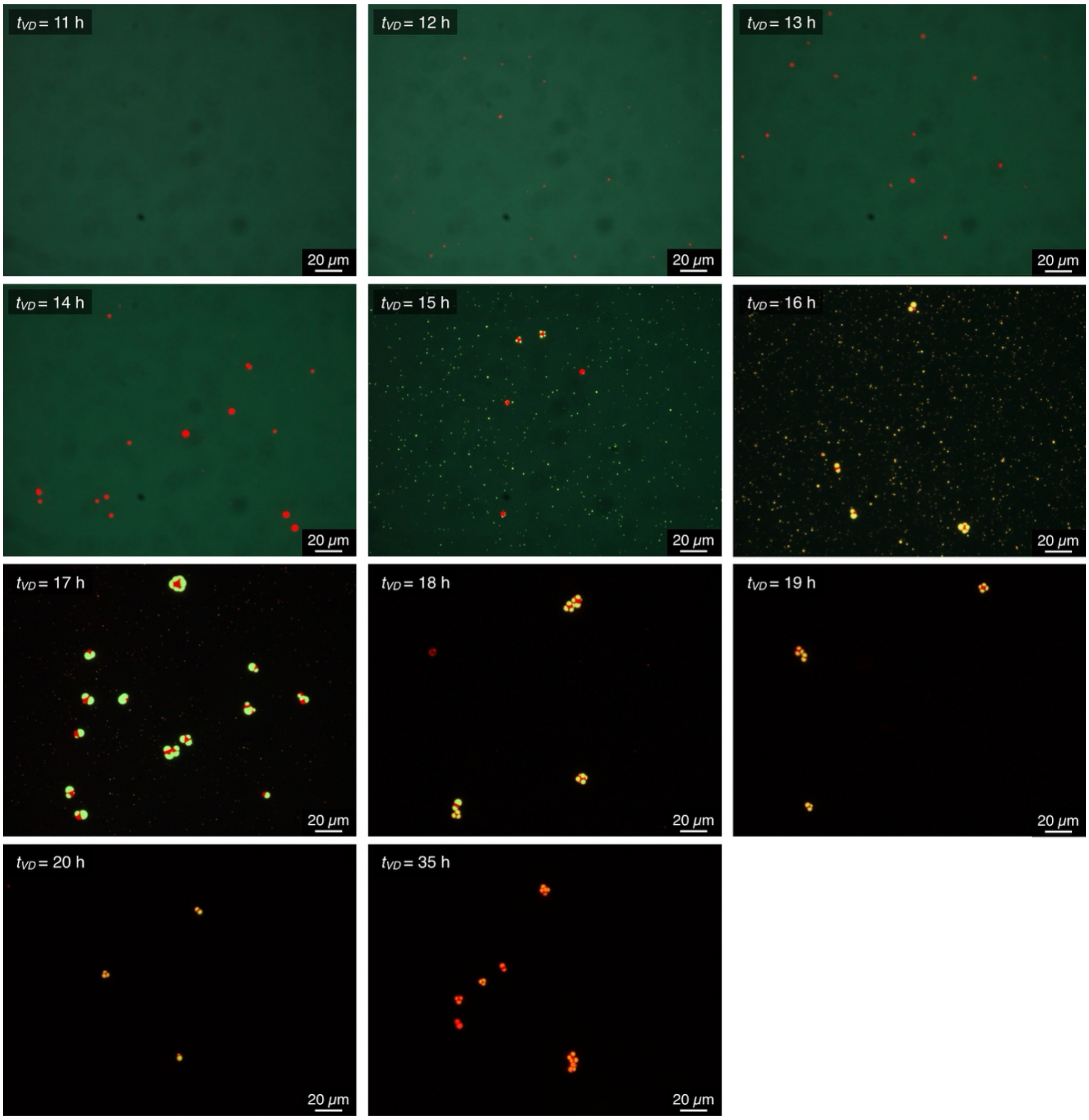

Figure S8. Time-course fluorescent micrographs of the coassembly of **P1**$_{22k}$/**P2** with $f_{\mathbf{P2}}$ = 0.4. λ_{ex} = 400–440 nm. The appearance of irregular patchy droplets at t_{VD} = 17 h is probably due to accidental mechanical deformation caused by uptake by the syringe or crush within the cover glasses.

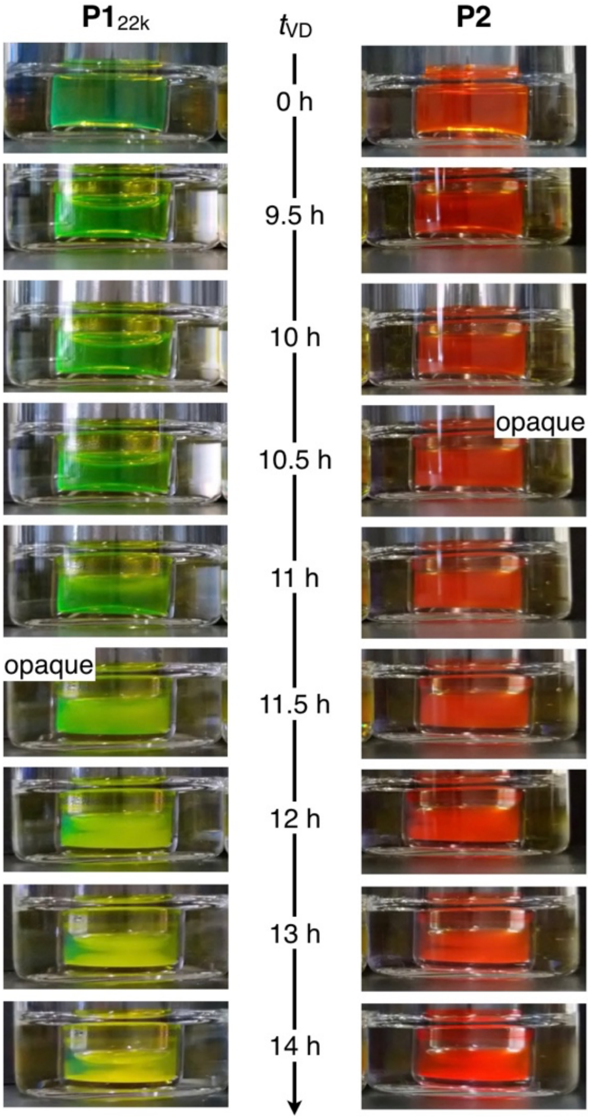

Figure S9. Time-course photographs of the vial of the homotropic assembly of **P1**$_{22k}$ and **P2** through the VD process. Each vial of **P1**$_{22k}$ and **P2** turns into opaque completely at t_{VD} = 11.5 h and t_{VD} = 10.5 h, respectively, indicative of poorer solubility of **P2** compared with **P1**$_{22k}$ for diffusing of MeOH.

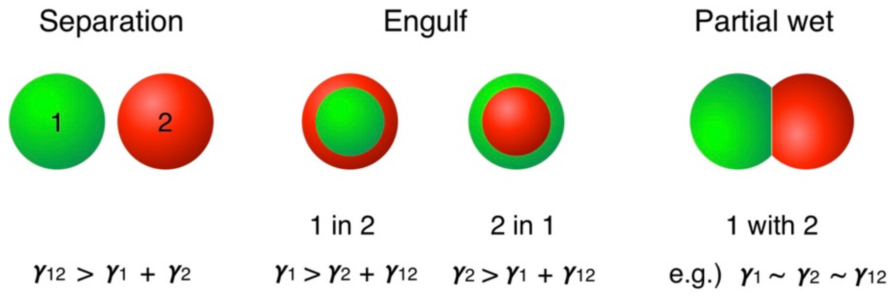

Figure S10. Schematic representations of possible structures of two droplets. Here, γ_1, γ_2, and γ_{12} represent the interfacial tensions between the **P1** droplet and the dilute phase (surrounding solution), the **P2** droplet and the dilute phase, and the **P1** droplet and the **P2** droplet, respectively.

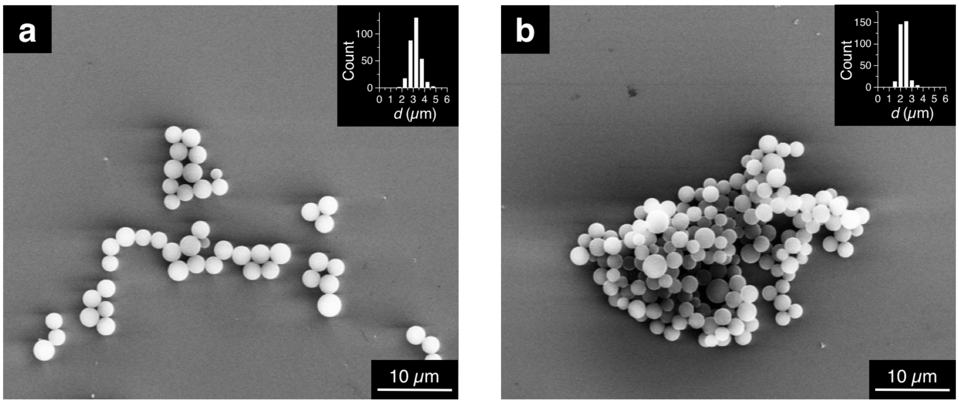

Figure S11. (a, b) SEM micrographs of self-assembled microstructures of **P1**$_{34k}$ (a) and **P1**$_{65k}$ (b). Insets show histograms of the diameter *d*.

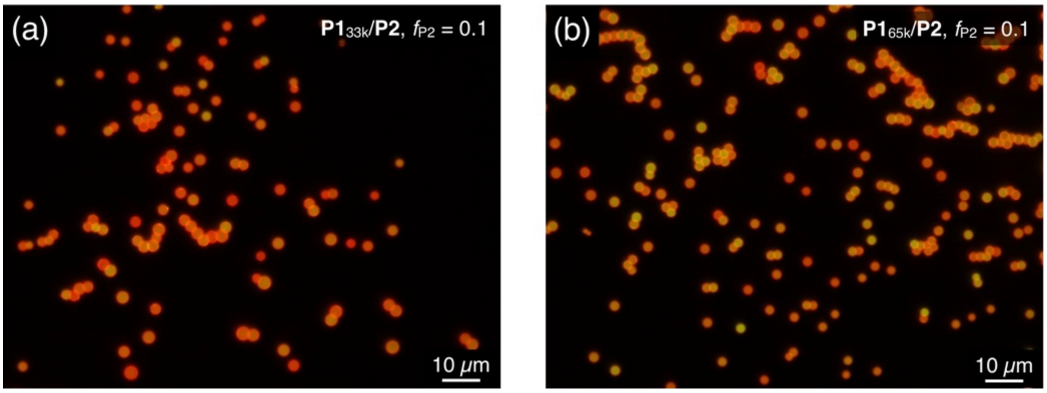

Figure S12. FL micrographs of coassembled microstructures of **P1**$_{34k}$/**P2** (a) and **P1**$_{65k}$/**P2** (b) with $f_{\mathbf{P2}}$ = 0.1. λ_{ex} = 400–440 nm.

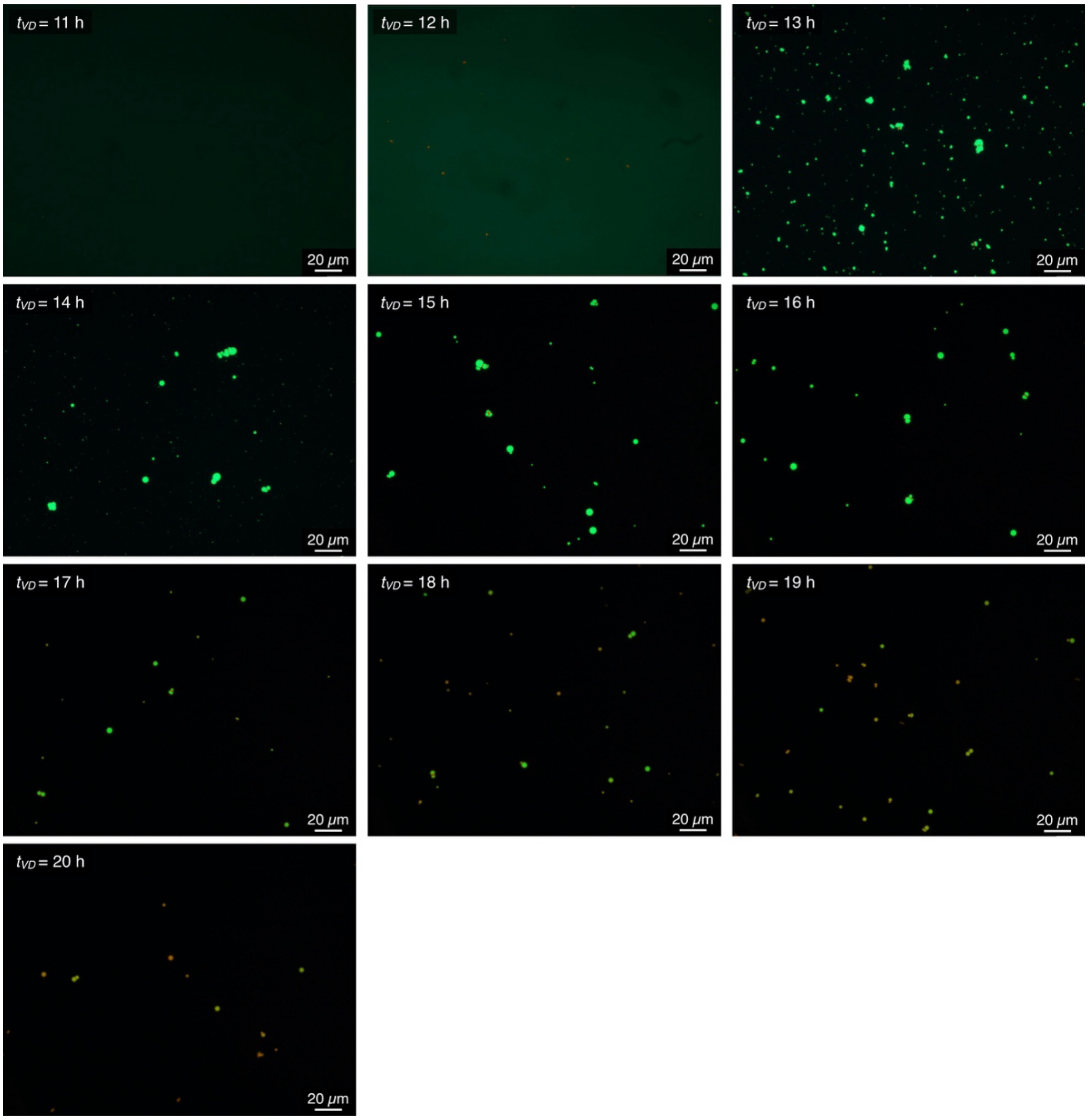

Figure S13. Time-course fluorescent micrographs of coassembly of **P1**$_{65k}$/**P2** with $f_{\mathbf{P2}}$ = 0.1. λ_{ex} = 400–440 nm. The appearance of irregular patchy droplets is probably due to accidental mechanical deformation caused by uptake by the syringe or crush within the cover glasses.

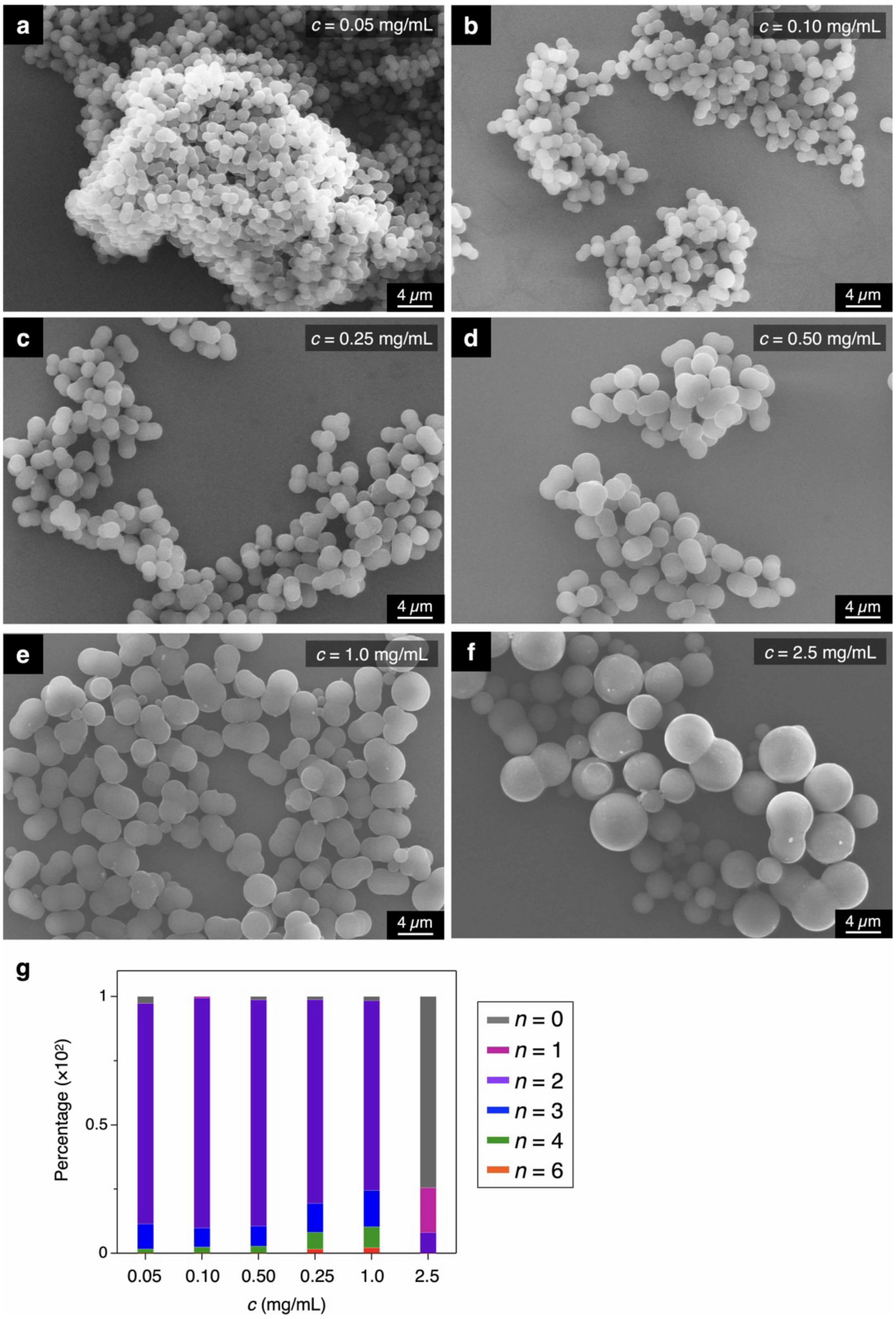

Figure S14. (a-f) SEM micrographs of coassembly of **P1**$_{22k}$/**P2** at $f_{\mathbf{P2}}$ = 0.3 with c = 2.5 (a), 1.0 (b), 0.5 (c), 0.25 (d), 0.1 (e), 0.05 (f). All SEM images were taken at the same magnification (×1000). (g) Bar chart, showing the percentage of the multilobe CMs with n = 0 (grey), 1 (magenta), 2 (purple), 3 (blue), 4 (green), 6 (orange) for f_{P2} = 0.3 by coassembly of **P1**$_{22k}$/**P2**.

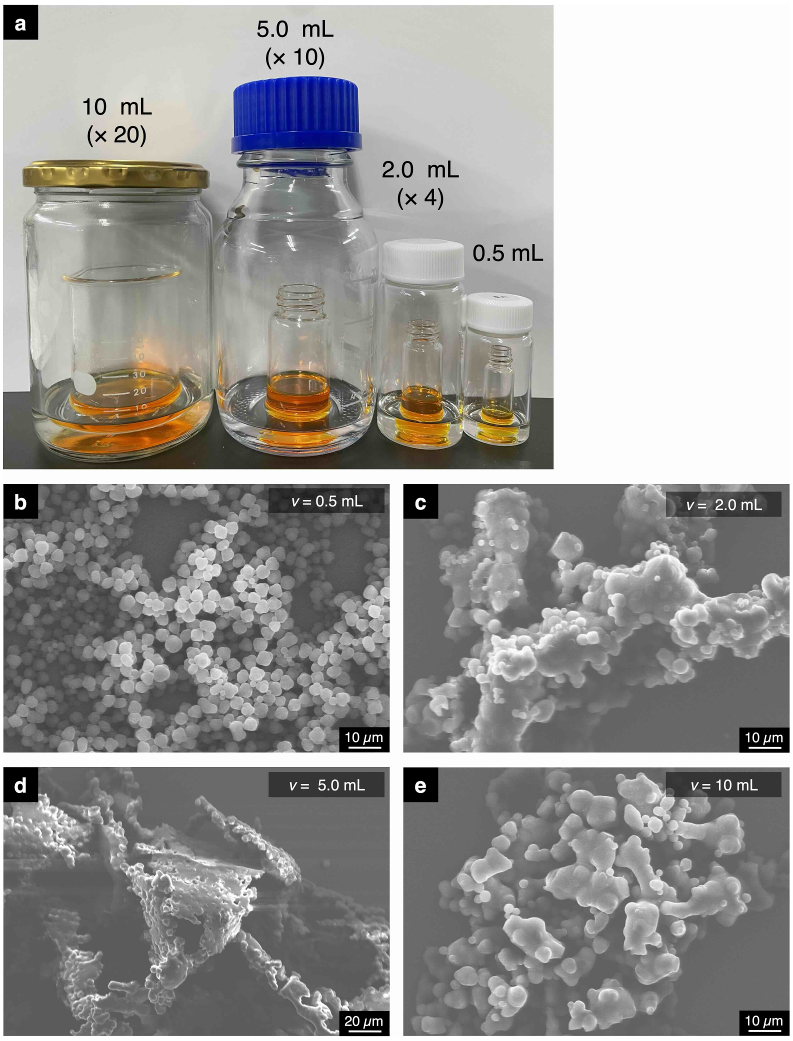

Figure S15. (a) Photograph of large-scale coassembly of **P1**$_{31k}$ and **P2** at $f_{\mathbf{P2}}$ = 0.4 ([**P1**$_{31k}$ /**P2**] = 0.5 mg mL^{-1}) with different volume scale (0.5 mL, 2.0 mL, 5.0 mL, and 10 mL of the polymer blend solution from right to left). (b-e) SEM micrographs of the resultant precipitates obtained from the polymer blend solution with the amount of 0.5 (b), 2.0 (c), 5.0 (d), and 10 mL (e).

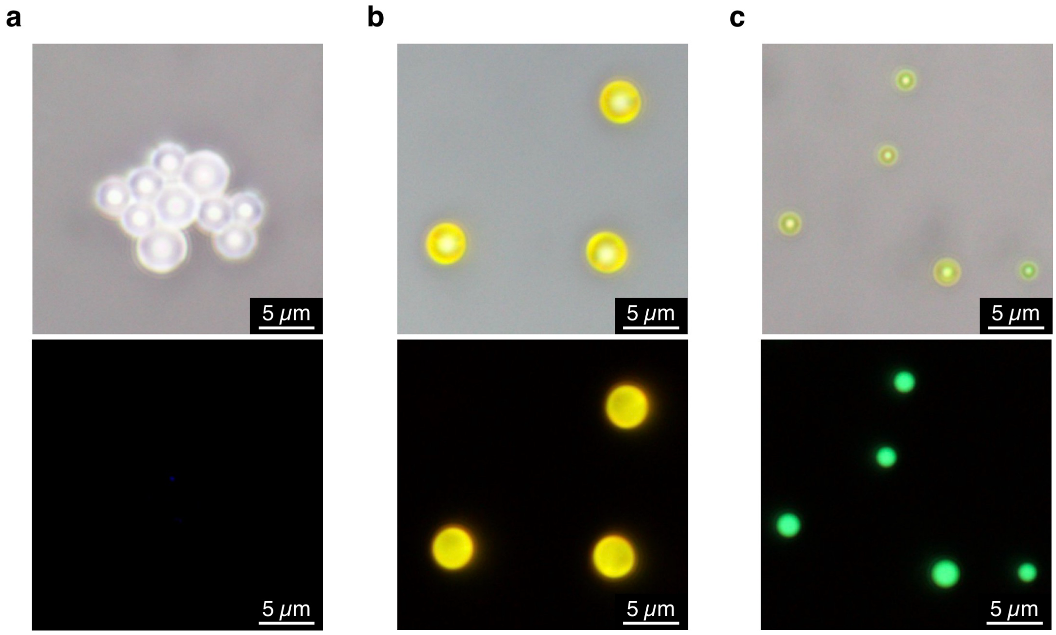

Figure S16. (a–c) OM (top) and FL (bottom) micrographs of self-assembled microspheres of **P3** (a), **P4** (b), and **P5** (c). For the FL micrographs, λ_{ex} is 340–390 nm (a), 400–440 nm (b), and 400–440 nm (c), respectively.

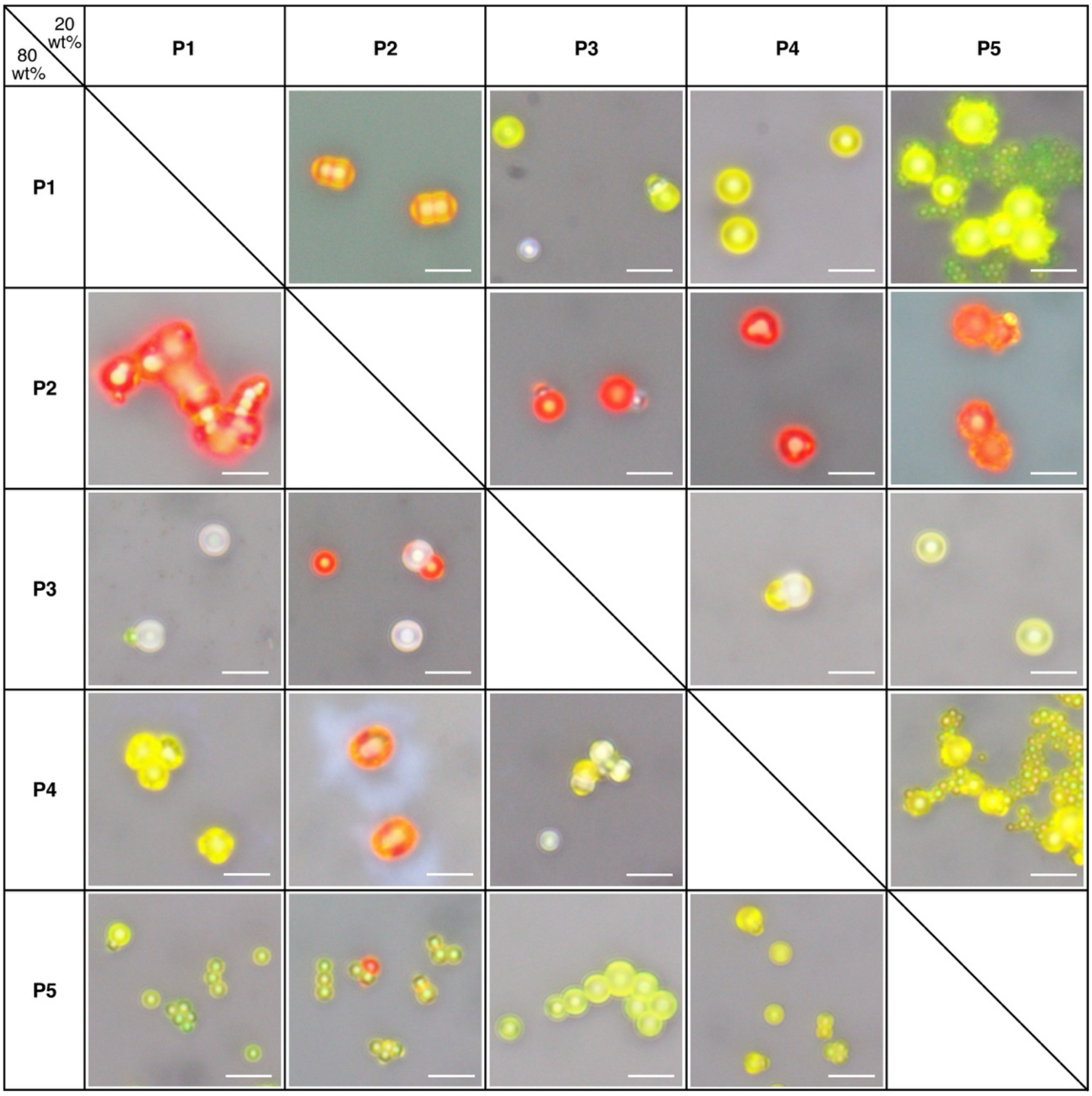

Figure S17. OM micrographs of coassembled microstructures prepared from binary polymer blends from **P1**$_{22k}$, **P2**, **P3**, **P4**, **P5**. For each blend combination, coassembly was performed with a weight ratio of 8/2 and 2/8 of one polymer to the other. Scale bar: 5 μm.

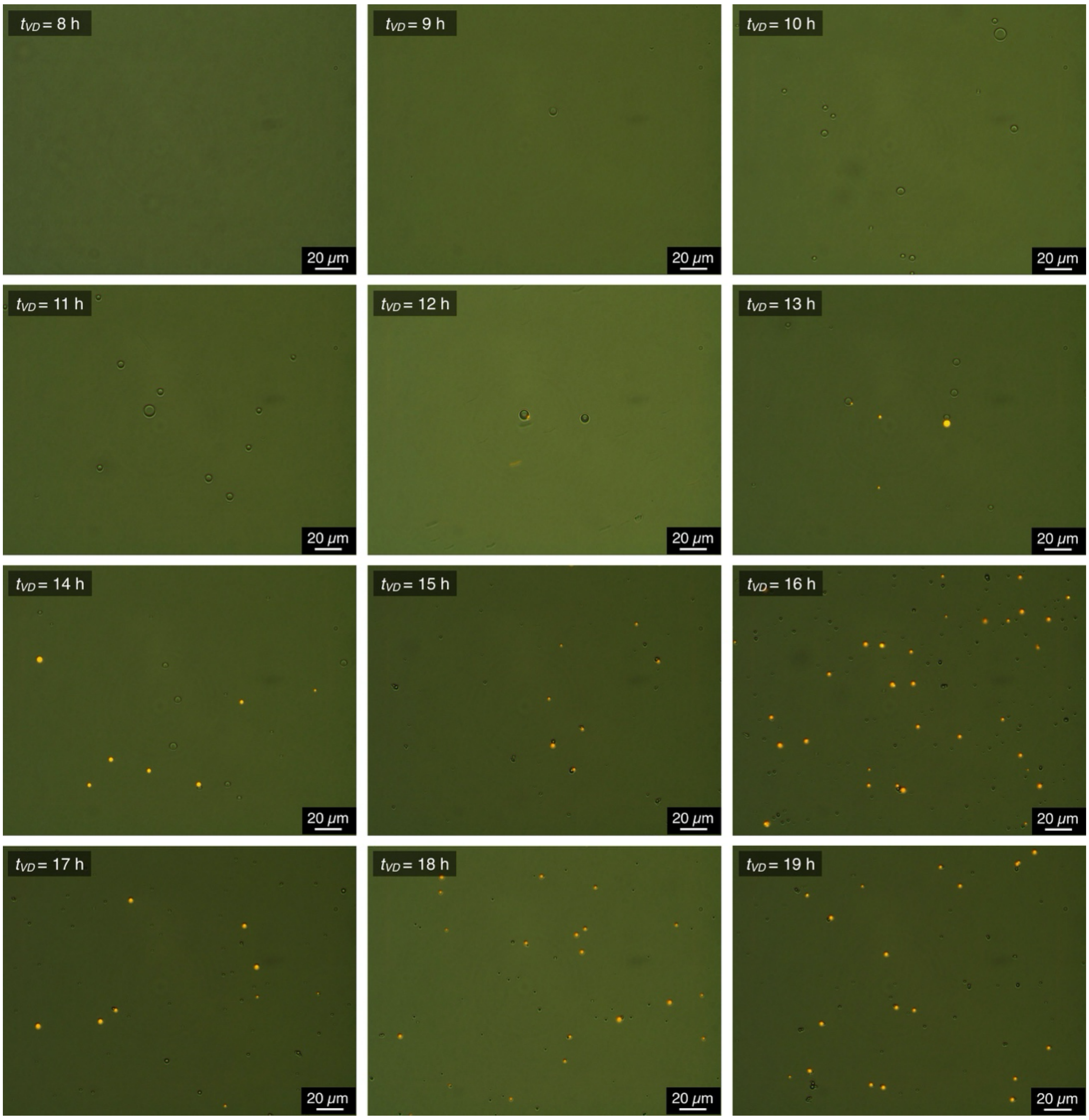

Figure S18. Time-course fluorescent micrographs of coassembly of **P3**/**P2** with $f_{\mathbf{P2}} = 0.2$. Background color is not completely dark because not only excitation light for **P2** (λ_{ex} = 400–440 nm) but also weak white light is illuminated to visualize **P3** during the observations.

4. Supporting Movies

Movie S1.

Fluorescence microscopy observation of fusion of the colloidal droplets in the liquid circumstance and the subsequent deformation in the drying process. The sample solution was prepared by the coassembly of $\mathbf{P1}_{22k}$/**P2** with $f_{\mathbf{P2}}$ = 0.4 and corrected at t_{VD} = 15 h. λ_{ex} = 400–440 nm.

Movie S2.

Fluorescence microscopy observation of fusion of the colloidal droplets in the liquid circumstance. The sample solution was prepared by the coassembly of $\mathbf{P1}_{22k}$/**P2** with $f_{\mathbf{P2}}$ = 0.4 and corrected at t_{VD} = 15 h. λ_{ex} = 400–440 nm.

5. Supporting References

[S1] H. Saito, J. Kuwabara, T. Kanbara, Facile Synthesis of Fluorene-Based π-Conjugated Polymers via Sequential Bromination/ Direct Arylation Polycondensation. *J. Polym. Sci., Part A: Polym. Chem.* **2015**, *53*, 2198−2201.

[S2] H. Saito, J. Chen, J. Kuwabara, T. Yasuda, T. Kanbara, Facile one-pot access to π-conjugated polymers via sequential bromination/direct arylation polycondensation. *Polym. Chem.* **2017**, *8*, 3006.

[S3] O. Oki, C. Kulkarni, H. Yamagishi, S. C. J. Meskers, Z.-H. Lin, J.-S. Huang, E. W. Meijer, Y. Yamamoto, Robust Angular Anisotropy of Circularly Polarized Luminescence from a Single Twisted-Bipolar Polymeric Microsphere. *J. Am. Chem. Soc.* **2021**, *143*, 8772–8779.

[S4] P. M. Budd, B. S. Ghanem, S. Makhseed, N. B. McKeown, K. J. Msayib, C. E. Tattershall, Polymers of intrinsic microporosity (PIMs): robust, solution-processable, organic nanoporous materials. *Chem. Commun.* **2004**, *2*, 230-231.

[S5] K. Zhou, J. G. Liu, M. G. Li, X. H. Yu, R. B. Xing, Y. C. Han, Phase Diagram of Conjugated Polymer Blend P3HT/PF12TBT and the Morphology-Dependent Photovoltaic Performance. *J. Phys. Chem. C* **2015**, *119*, 1729–1736.

[S6] J. Burke, Solubility Parameters: Theory and Application; The Book and Paper Group of the American Institute for Conservation, 1984.

[S7] L. Tao, L. Lihui, C. Dingfu, S. Haoran, W. Jiaqian, C. Yuhui, C. Hao, S. Wei, C. Kun, C. Shufen, Efficiency enhancement of organic light-emitting diodes via a self-assembled moth-eye nanopatterned hole-transporting layer. *Optics Express* **2021**, *29.5*, 7210–7219.

[S8] S. Nilsson, A. Bernasik, A. Budkowski, E. Moons, Morphology and Phase Segregation of Spin-Casted Films of Polyfluorene/PCBM Blends. *Macromolecules* **2007**, *40*, 8291–8301.
